# Opening up while locking down: how an Irish independent sector mental health service is responding to the COVID-19 crisis

**DOI:** 10.1017/ipm.2020.68

**Published:** 2020-05-27

**Authors:** Paul Fearon

**Affiliations:** 1 St Patrick’s Mental Health Services, Dublin, Ireland; 2 Department of Psychiatry, Trinity College, University of Dublin, Dublin, Ireland

**Keywords:** COVID-19, mental health service, strategy, safety

## Abstract

The COVID-19 pandemic poses a particular set of challenges for health services. Some of these are common across all services (e.g. strategies to minimise infections; timely testing for patients and staff; and sourcing appropriate personal protective equipment (PPE)) and some are specific to mental health services (e.g. how to access general medical services quickly; how to safely deliver a service that traditionally depends on intensive face to face contact; how to isolate someone who does not wish to do so; and how to source sufficient PPE in the face of competing demands for such equipment). This paper describes how St Patrick’s Mental Health Services (SPMHS) chose to address this unfolding and ever-changing crisis, how it developed its strategy early based on a clear set of objectives and how it adapted (and continues to adapt) to the constantly evolving COVID-19 landscape.

## Introduction

St Patrick’s Mental Health Services (SPMHS) is the largest independent provider of mental health services in Ireland. It consists of three inpatient approved centres: St Patrick’s University Hospital (SPUH), Dublin 8, which comprises 241 adult beds; Willow Grove Adolescent Unit (WGAU), a 14-bedded approved centre on the SPUH site; and St Edmundsbury Hospital (SEH), Lucan, a 52-bedded adult approved centre. The service provides a wide range of mental health specialties, including general adult psychiatry, psychiatry of old age, addictions and dual diagnosis, eating disorders, anxiety disorders, a young adult service, the largest electroconvulsive therapy (ECT) service in Ireland and a 12-bedded acute intensive care ward. Thus, it accounts for approximately 8% of general adult beds nationally. SPMHS also offers a wide range of day programmes, which are accessed internally or via direct primary care referral, and a network of outpatient and community-based clinics (‘Dean Clinics’) in Dublin 8, Lucan, Sandyford, Cork and Galway. Annually, we see over 1000 new assessments and over 15 000 reviews in our clinics.

Operating in the independent sector, the service receives no government funding but instead relies largely on funding via the three major health insurers in Ireland. It also has various arrangements in place with some Health Service Executive (HSE; publicly funded) Community Healthcare Organisations to provide services when required, most commonly in the areas of child and adolescent psychiatry, eating disorders and the provision of ECT. End-of-year surpluses are very modest and are reinvested within the organisation to continually improve and expand the service. The organisation employs 700 staff.

The following is an account of how our service chose to respond to unfolding events and the lessons we are learning as a result. At the time of writing (16–19 April 2020), we have no confirmed cases of COVID-19 infection among our inpatients (we previously had one mildly symptomatic case who has completed their self-isolation) and have had two staff members who tested positive (both of whom have completed their self-isolation). Our 14-bed adolescent unit is full and our adult bed occupancy is currently 86% (253 inpatients). The same day last year, our adolescent unit had 13 inpatients and our adult occupancy was 96% (281 inpatients).

## Prediction

In mid-February 2020, it was becoming increasingly clear from events in both China and Italy that COVID-19 was likely to become a global issue requiring both national and international action. Ireland recorded its first case of COVID-19 infection on 29 February (Irish Times, March 2, 2020). Four days later, there were six confirmed cases, including a family of four recently returned from Italy.

Even at an early stage, there was already enough information available to answer the following questions: Will this virus arrive in Ireland? Will it follow a similar pattern of transmission as had already occurred in China and Italy if left unchecked? Is it likely that there will be an effective vaccine or treatment prior to a surge in cases? If not, what are the likely initial measures necessary to protect society and reduce the risk of the virus spreading uncontrollably? What measures are China and Italy adopting? The questions may have been challenging and unprecedented, but the answers, even in mid-February before the first confirmed Irish case, appeared relatively obvious. Addressing these questions early and objectively allowed us to formulate an initial strategy, informed by what we felt needed to be our main aims in responding to the unfolding crisis and, crucially, allowed us to stay just a day or two ahead of the crisis as it gathered pace.

On 26 February (3 days before the first confirmed Irish case), we held our first meeting, attended by the Medical Director, Director of Nursing and Director of Services, concerning this new virus and to explore how we might respond to it. Many further meetings followed over the next 2 weeks and a core ‘COVID-19 Response Team’ emerged, chaired by the CEO and comprising the Medical Director, Director of Nursing, Director of Services, Head of IT, Chief Financial Officer, Head of Procurement, Director of Digital Transformation and Head of Communications and Clinical Governance Officer, among others. From the start, these meetings were intensely focused on the immediate issues with a view to agreeing pragmatic solutions or courses of action by the time the meeting (which rarely lasted more than 30 minutes) was over.

Our agreed aims guiding our initial strategy were (1) to protect our service users and staff from contracting the virus to the best of our ability and by following national guidelines, (2) to treat as many people requiring mental health interventions as we could, always within a human rights-based ethos, and (3) to remain open and viable as an organisation so that we were in a position to assist with the almost certain increased demand for mental health care nationally during and in the aftermath of the projected pandemic.

## Strategy formulation

We quickly agreed that it was highly likely that Ireland would, like other countries, suffer a significant outbreak of COVID-19. It seemed certain that the most likely measures to attempt to limit person-to-person spread would involve reduced physical contact and other similar restrictions. These reasonable assumptions allowed us to conclude that, in a mental health service setting, the main challenges would involve making timely decisions controlling access to our campuses while at the same time finding ways to continue to offer our services to those who required it, quickly limiting staff footfall to only those who could not work remotely, determining how we would manage confirmed or suspected cases among service users and staff if they arose, and actively exploring the extent to which we could safely deliver care to our service users by remote means. We needed to address these issues while at the same time bearing in mind Mental Health Commission (MHC) regulations and standards, the human rights of service users and staff, and any legal issues.

## Actions

Having formulated our priorities, we worked swiftly to address them in several, often overlapping, domains. These are described next in this section.

### Access

We were quick to move towards limiting any unnecessary footfall into and out of our various sites. Initially, weekend therapeutic leave was curtailed unless there were exceptional circumstances. Visiting was restricted to one visit per service user per week and service users were required to inform ward staff, 1 day in advance, of a visitor that they were expecting. Visits occurred in a designated area. A daily visitor list was compiled and distributed to reception staff and inpatients were asked to inform potential visitors of our new measures (in addition to publishing this information on our website). Only those on the daily list were permitted access to our buildings. External events moved at such a pace that when we devised this visitor policy, we wondered if it would be viewed as an overreaction to events. As it happened, on the first day of its introduction, the Irish Government announced the first in a series of social distancing measures, which suddenly rendered our policy less radical than it had appeared only hours previously. Only a week later, in response to further social distancing measures nationally, all physical visiting ceased. In tandem with this, our IT department rapidly developed protocols to allow inpatients to securely communicate with their families using their own devices and also procured a sufficient number of devices for use by those without such technology to hand. A number of social and recreational activities remained in place, adapted to comply with physical distancing, but all therapeutic inpatient groups rapidly moved to remote access only. These measures were largely understood and supported by inpatients who realised, even at the early stages of the virus outbreak, that our rationale was to protect them while continuing to offer them a comprehensive service.

Other routes of access were examined and protocols drawn up to minimise the introduction of infection. Areas such as daily deliveries, the ingress and egress of staff and external groups who held weekly meetings in our campus, outpatient and day service groups were either altered, cancelled or moved to remote access only.

Almost all of our HR, finance, IT, administration and procurement staff have been working remotely for several weeks now but are fulfilling their responsibilities seamlessly and are only a phone or video call away. In addition, many clinicians (the majority of Allied Health professionals and some medical and nursing staff) are successfully working remotely.

### Technology

There were two major areas where technology was likely to play a major role in our response to COVID-19. The first was our Electronic Health Record (EHR) system that we had developed over 3 years and which went live in September 2017. This immediately gave us the means for clinicians to work remotely if required while having up to date access to a service user’s full clinical record, including the ability to add notes, to update risk ratings and care plans and to prescribe medication. It also eliminated the need for staff to retrieve and return paper records (with the attendant risk of infection).

The second major role for technology lay in the realm of video-enabled working. Our organisation already had some experience in this area, having successfully commenced video-enabled Cognitive Behavioural Therapy (CBT) sessions some months previously and being in the process of piloting such technology in the child and adolescent service. We determined early in the crisis that we needed to move as much work as quickly and safely as was possible to this medium so that we were familiar with it before it became absolutely essential. Thus, most of us moved to remote meetings in early March. All meetings have been held using remote technology in our organisation for the past 5 weeks and all staff are now both familiar and comfortable with it. Clinically, weekly team multidisciplinary meetings are routinely held remotely, as are individual appointments with allied health professionals, and several elements of ward rounds are also delivered in this fashion. Our IT department looked at the various platforms and determined that in terms of security, ease of use, reliability and convenience, Microsoft Teams (Microsoft, 2020) was the preferred platform for our organisation. This was uploaded to all staff’s computers along with an instruction guide.

Our IT department has played a key role in enabling this rapid transformation, by ensuring that we have a sufficient number of suitably equipped electronic devices for both staff and service users. It has also developed a specific Service User IT service (‘SUITS’) to respond to service user queries about the new technology.

### Remote treatment options

Consistent with the measures outlined above to increase social distancing while at the same time continuing to provide a comprehensive service, we examined all of our treatment options. We moved swiftly to convert all outpatient appointments to video or (if video was not feasible) telephone consultations. By the week of 23rd March, all outpatient appointments were being delivered remotely. Each week since then, between 240 and 301 appointments have taken place, which constitutes between 70% and 90% of our expected level of activity compared to the same time last year. This comprises both new assessments and review appointments. To date, there has been no particular group (clinically or demographically) for whom this option has been a significant barrier.

Similarly, day and inpatient programmes were rapidly modified to allow them to be delivered exclusively by remote technology. The first-day programme was delivered remotely on 20th March and all-day programmes (both general and disorder-specific) have been running in this way since 30th March. The percentage of attendees on these programmes is 69% compared to the same period last year. In making the transition to remote delivery, we have had to modify these programmes to some degree, but they remain fully interactive, with participants being able to log in at the scheduled time, to interact with the clinician leading the group and with other participants, and to access any information sheets via email.

Perhaps the most radical and critical change to our service delivery options was the introduction of Extended Therapeutic Leave (allowing inpatients to complete the remainder of their inpatient treatment at home via up to a maximum of 13 days) and Home Care services (allowing patients deemed suitable the option of experiencing their full inpatient treatment programme, from admission to discharge, from home) for the delivery of inpatient-level care to people in their homes via remote technology. In brief, we proposed to the three main health insurers that, due to the unprecedented nature of the COVID-19 crisis, it was vital that we explore options that allowed us to deliver, to those who were deemed clinically suitable, the same interventions remotely that they would receive if they were a physically present inpatient. These included a full psychiatric admission, creation of an initial care plan, the prescription of medication (faxed to their local pharmacy and reimbursed by us), daily contact by members of the Multidisciplinary Team (MDT), including ward nursing staff, consultant-led ward rounds, weekly MDT meetings, assessment for and attendance at appropriate therapeutic groups, and assessment and treatment as appropriate by allied Health professionals (e.g. psychology, CBT, social work and occupational therapy).

These new services involved a leap of faith and trust for all parties concerned (our organisation, service users and health insurers) but all three main insurers understood the need for such measures in the current climate and approved them, showing immense courage and foresight. We retain clinical responsibility for those on special inpatient treatment packages and in all cases reserve a physical bed on site to ensure that we treat the number of patients that we have the capacity to treat. This additionally gives each patient the reassurance that, should they need to return to or present at our hospital at short notice, there is a bed reserved specifically for them. At the time of writing, we have 93 inpatients currently being treated via remote means in their homes and the informal feedback from both patients and staff has been largely positive. In general, the criteria for suitability for either of the above remote services are (1) the individual agreeing to this mode of service delivery and (2) being deemed clinically appropriate by their current or prospective treating team in terms of risk management and being satisfied that all essential elements of treatment can be delivered safely remotely.

### Using two sites rationally

We have had only one confirmed case of COVID-19 in an inpatient in our service. Although this patient was only mildly symptomatic and able to complete his 14-day self-isolation period, the additional need for his close contacts to self-isolate brought home to us how vulnerable each ward was if even one case was identified. We chose to take advantage of the fact that we have two adult approved centres in geographically separate locations (SPUH and SEH) and, after working carefully on the logistics, transformed SEH into our centre for people required to self-isolate due to COVID-19, and retained SPUH as a COVID-free zone. Thus, confirmed or suspected COVID-19 cases or those who, for whatever reason, needed to self-isolate, could move to SEH to complete this process (and return to SPUH thereafter) while continuing their mental health treatment, while allowing SPUH to continue to operate as a COVID-free centre. This has worked well to date. We have had between 2 and 12 people in SEH at any one time (we have 3 at the time of writing). This also allows a cohort of nurses to build up expertise in working with people with confirmed or suspected COVID-19 infection.

### Admissions

In order to keep SPUH a COVID-free site while accepting as many admissions as we can, we have developed a protocol for the timely assessment of COVID risk. Referrers are asked to complete a short COVID-status questionnaire and a brief COVID underlying risk factors form (both based on national guidelines) so that we can assess and most appropriately place any admission, once accepted by a clinical team. We remain open to referrals from any source and, to date, have not declined any admission on the basis of COVID risk. Our admissions triage team, chaired by the Medical Director and comprising the Director of Nursing and Director of Services, meets daily at 03:00 pm to review all referrals for the following day in order to ensure at a service level that all admissions are carefully and appropriately accommodated.

### Staff

Once issues relating to admissions, inpatients and deliveries had been addressed, it was clear that the remaining major source of potential infection comes from staff onsite who come and go daily. Early in the crisis, we closed the hospital gym (initially prohibiting staff for this reason) and introduced strict physical distancing measures in the hospital restaurant (staff only, one person per table, tables 2–3 metres apart). We also employed an external Occupational Health Service to give objective advice to staff who were either ill or symptomatic (related or unrelated to COVID-19) and who followed national guidelines closely. We have always followed and supported this advice without question.

### Prioritising services and equipment

In early March, we formed the view that personal protective equipment (PPE) would be in short supply and would likely, at least initially, be prioritised for general hospitals. We were clear from the start that we would follow all national guidelines relating to the management of suspected or confirmed cases and the appropriate use of PPE (and have continued to do so without exception) (Health Protection Surveillance Centre, 2020). We also knew that we needed to identify key services that were either essential or had specific additional COVID-19 vulnerabilities. Thus, while all areas within our organisation have sufficient and appropriate PPE, we continue to particularly ensure that our SEH site, our ward for elderly patients, our ECT suite (Colbert *et al*. 2020) and our Special Care Unit are always sufficiently stocked with the PPE appropriate to their function (and in line with national guidelines). We source modest amounts of PPE from a wide variety of sources and have regular inventory checks, which allow us to plan for the best and worst case scenarios in terms of PPE usage. Although our supply is never at a completely comfortable level, we have to date not fallen critically short.

### Shared leadership and communication

The rapid, at times breathtaking, pace with which services have had to adapt to the challenges around COVID-19 has posed particular issues around decision-making and the effective communication of those decisions. As an independent sector service, we may be fortunate in that we are used to being agile in our decision-making processes and that a culture of shared leadership permeates the organisation. This has allowed us to consider issues carefully, and then, crucially, to act on our decisions without undue delay. Central to this process is effective and rapid communication to those affected by these decisions. Equally vital is the contribution by all members of staff and our service users. These rapid changes and innovations would not have been possible to implement successfully without the dedication, hard work, good will and flexibility of all of these individuals.

We have worked hard to ensure that all patients are informed of any changes to our services (and the rationale underpinning them) as soon as they are agreed upon. Further, a letter was issued to all GPs in Ireland on 24 March informing them about our contingency measures and the new remote services available for inpatients, outpatients and day patients. Emails were also sent to HSE Business Managers and GPs on our digital network, informing them of our willingness and ability to consider their referrals.

Rarely a day goes by when I don’t email (or meet remotely) several other senior management, consultant and registrar colleagues to keep them up to date on what is happening or what may be about to happen. Trust in one’s colleagues in these times is arguably the most important currency and needs to be cherished and nurtured.

### Recuperation

This crisis is almost certainly going to continue in some form for several months or more. As we move from one phase to the next, we need to pace ourselves when possible. We are all tired and there seems to be no break from having to adapt to constant change. Equally, when there is a brief lull for a day or two, a small space opens up allowing us to realise how truly exhausted we are. In this context, I have been encouraging our medical staff to take their annual leave as scheduled. Some have asked to postpone it for a variety of reasons and some have returned early, but I believe it’s important that we all try to take time to recuperate where possible, or at least know that the opportunity is always there to do so. This is a marathon, not a 5k.

## The future

It seems highly unlikely that when this pandemic is finally over or under control, things will return to the way they were. Our ways of working will almost certainly change. Initiatives that would have been unthinkable 2 months ago are now mainstream. Treatment delivery options that would normally have been piloted over a period of months or more are now in use daily. It will be vital to carefully and objectively evaluate all of these initiatives to determine use in the longer term, particularly in a post-COVID world. We are currently surveying all of our service users who are receiving any of our remote treatment options to more formally evaluate their acceptability and effectiveness.

Although the rapid introduction of video-enabled remote service delivery by many services was a necessary response to the current crisis, I believe that this clinical service delivery modality will remain relevant after COVID-19 is under control or eliminated. One of the more interesting facets of this is that it makes the argument as to whether inpatient or community care is superior somewhat redundant. The truth is (and always was) that neither is superior. The best treatment is the one that is most appropriate for each individual at a particular point in time and delivered to a high standard and properly resourced. Video-enabled services, delivered to a high quality, have the potential to increase the treatment options we can offer our service users. It may soon be time to have an updated vision for A Vision for Change (Expert Group on Mental Health Policy, 2006), incorporating what we are learning, and will continue to learn, from this crisis.

It is almost certain that there will be a surge in the need for mental health interventions over the next year (Galea *et al*. 2020). Psychiatry as a discipline needs to be forthright, united and resolute in calling for the resources to meet this need without delay so that this curve, too, can be flattened by addressing it early. This is the time, if ever there was one, where we need to work together, share our experiences in this new and challenging environment and lobby effectively for the resources to respond to the mental health needs of our fellow citizens.
